# A psychometric analysis for the adaptation of the comprehensive breast cancer knowledge test for the male population: a methodological study

**DOI:** 10.3389/fpubh.2025.1535564

**Published:** 2025-07-23

**Authors:** Dercan Gencbas, Serpil Ozdemir, Leyla Yaman Uzumcu, Yunus Emre Bulut

**Affiliations:** ^1^Department of Public Health Nursing, Gulhane Faculty of Nursing, University of Health Sciences, Ankara, Türkiye; ^2^Department of Public Health, Gulhane Faculty of Medicine, University of Health Sciences, Ankara, Türkiye

**Keywords:** breast cancer, breast screening, female, male, reliability, validity

## Abstract

**Objective:**

Increasing men’s knowledge and awareness of breast cancer is aimed at addressing a significant barrier to women’s engagement in screening. This study aims to adapt the Breast Cancer Knowledge Test, originally designed for women, for use with men, in order to increase their awareness and support female screening efforts.

**Methods:**

This methodological study was conducted with 310 male volunteers. The Davis technique was utilized to evaluate content validity to adapt the Breast Cancer Knowledge Test for males. The construct validity of the scale was evaluated using Tetrachoric Factor Analysis. Kuder Richardson-20 was calculated to determine the reliability of the scale. Item difficulty and discrimination indices were tested using Rasch analysis.

**Results:**

The Content Validity Index value was 0.96. The data was suitable for the factor analysis according to Bartlett’s statistics (*p* = 0.001) and Kaiser-Meyer-Olkin test (test = 0.92). In the tetrachoric factor analysis, there were two subscales, explaining the total variance of 60.94%, and the goodness of fit indices were evaluated as indicating “excellent fit.” According to the Rasch analysis, the model was found to be significant (*p* < 0.05), and the infit and outfit values were within the range of 0.5 to 1.5.

**Conclusion:**

The male version of the Breast Cancer Knowledge Test was a valid and reliable tool for assessing the knowledge level of the Turkish male population regarding female breast cancer. By identifying areas where men lack knowledge, this scale can inform the development of targeted public health and educational initiatives, ultimately improving male engagement in breast cancer awareness and supporting female screening efforts.

## 1 Introduction

Breast cancer is the most commonly diagnosed cancer among women worldwide. In 2020, over 2.3 million women were diagnosed with breast cancer worldwide (1), and it is reported that breast cancer accounts for 15.5% of cancer-related deaths (2). Early detection of breast cancer significantly increases the effectiveness of treatment, thereby reducing mortality rates associated with the disease. Recommended practices for the early detection of breast cancer include breast self-examination, clinical breast examination, and mammography screening (1, 3, 4). Among these, regular mammography screening has proven to be highly effective in early detection and reducing mortality rates (5–7). However, the literature frequently emphasizes that breast cancer awareness, as well as knowledge of risk factors and early detection methods, is essential for the effective and widespread implementation of screening programs (7–12). To significantly reduce the mortality associated with breast cancer, at least 70% of the at-risk population should participate in mammography screening (13). In this context, both international and national authorities encourage women to engage in breast cancer prevention, early detection, and screening programs (3, 14, 15). Globally, participation rates in breast cancer screening programs remain below the desired level, with Northern European countries achieving rates over 80%, while other countries such as Italy (61.3%), Turkey (26–52%), Brazil (12–31%), Malaysia (7–25%), and China (22.5%) report much lower rates (16–22).

Several barriers hinder women’s participation in screening programs, including discomfort with screening procedures, lack of information, stigma and/or fear related to cancer, gender discrimination, inadequacies in social support systems, low socio-cultural and economic status, as well as negative health attitudes and beliefs towards screening (4, 15, 23, 24). Therefore, new strategies are needed to overcome these barriers and increase participation rates (3–25). The role of men in accessing reproductive health and cancer screening programs for women has been increasingly emphasized, and their involvement in strategies for cancer screening awareness has been encouraged globally in recent years. The literature also highlights the potential role of men in improving women’s participation in breast cancer screening programs, especially in patriarchal societies where men are often primary decision-makers regarding family health choices (4, 15, 24, 26, 27).

In many patriarchal societies, particularly in the Middle East, the socio-cultural expectation that women must obtain their husbands’ approval to participate in breast cancer screening represents a significant barrier (4, 23, 26). In Turkey, especially the eastern part, women face difficulties in making independent decisions regarding their health, as societal norms emphasize the authority of men within the family. This situation creates a barrier that hinders women from fully exercising their health rights, with the approval of male figures in the family taking precedence in health-related decisions (28). Men’s lack of knowledge and awareness about breast cancer, along with negative health attitudes and beliefs, could contribute to women’s exclusion from screening programs (26, 27, 29). Raising awareness among men, especially in patriarchal societies, could eliminate this barrier, allowing men to encourage the women in their families to participate in screening programs (30). However, to effectively play this role, it is essential to identify the extent of the knowledge gap among men and address it, as fostering a positive health attitude, behavior, and belief is crucial (4).

Despite the availability of tools to measure women’s knowledge of breast cancer (8, 31–33), no measurement tool specifically designed to assess men’s knowledge regarding breast cancer in women has been identified. One of the widely used, valid, and reliable instruments for measuring breast cancer knowledge among women is “The Comprehensive Breast Cancer Knowledge Test (BCKT),” developed by Stager (1993) (34–38). However, existing tools do not address the specific need for assessing men’s knowledge, particularly in societies where their awareness could significantly impact women’s participation in screening programs. This study aims to adapt the BCKT for the male population to fill this critical gap and enhance male involvement in breast cancer awareness and screening efforts.

## 2 Materials and methods

### 2.1 Study design and settings

This study was conducted using a methodological research design between April and August 2023 at a family health center. Given that this study involves adapting an existing tool, the Comprehensive Breast Cancer Knowledge Test (BCKT), to a new population (i.e., men), a methodological study design was selected that would allow for a systematic and rigorous adaptation process. The research took place at a family health center located in the city center of Ankara, the capital of Turkey. At this health center, outpatient diagnosis and treatment services, chronic disease care and monitoring, home care services, and the monitoring and care of at-risk groups (women, pregnant individuals, children, and the older adults) are provided by three doctors and four nurses.

### 2.2 Sample size and study population

The study sample consists of male individuals who visited the Family Health Center. The total number of registered males at the Family Health Center is approximately 3,100. For the sample size calculation in this study, the classification established by Comrey and Lee (39) for scale studies was utilized. Techniques such as factor analysis, they classify the sample size to enhance statistical power, ensure reliable estimation of model parameters, and analyze more variance while accurately evaluating this variance. According to their classification, a sample size is evaluated as follows: “50 participants = very poor,” “100 participants = poor,” “200 participants = adequate,” “300 participants = good,” “500 participants = very good,” and “1,000 participants = excellent.” Furthermore, advanced statistical techniques such as factor analysis or Rasch analysis require 10 participants per item to obtain accurate results and ensure reliable estimation of the model parameters (40). In this study, it was determined that a minimum sample size of 300 would be necessary for a “good” level of representation, and the study was completed with a voluntary sample of 310 men. Inclusion criteria for the study were: (i) being registered at the relevant family health center, (ii) being at least 18 years old, and (iii) having a mother and/or spouse who are alive. Individuals who were healthcare personnel or students in the health field, as well as those with a mother and/or spouse who were healthcare personnel, were excluded from the study.

### 2.3 Measures

Data for the study were collected using a demographic information form and the BCKT: male version. The demographic information form included 11 questions covering participants’ sociodemographic characteristics, such as age, education level, income status, and marital status (5 questions), as well as health characteristics related to the family history of cancer and breast cancer (6 questions) (4, 24, 26, 27, 30).

The BCKT adapted for males was developed by Stager (34) to measure women’s knowledge about breast cancer. The Turkish validity and reliability study of the scale was conducted by Basak (36). The scale consists of 20 items and two subscales. The general knowledge subscale includes the first 12 items (items 1–12), while the treatability subscale covers the last 8 items (items 13–20). Each item is marked as “Yes” or “No,” with correct answers coded as “1” and incorrect or unanswered responses coded as “0.”

#### 2.3.1 Adaptation of the breast cancer knowledge test: male version

**Figure 1:**
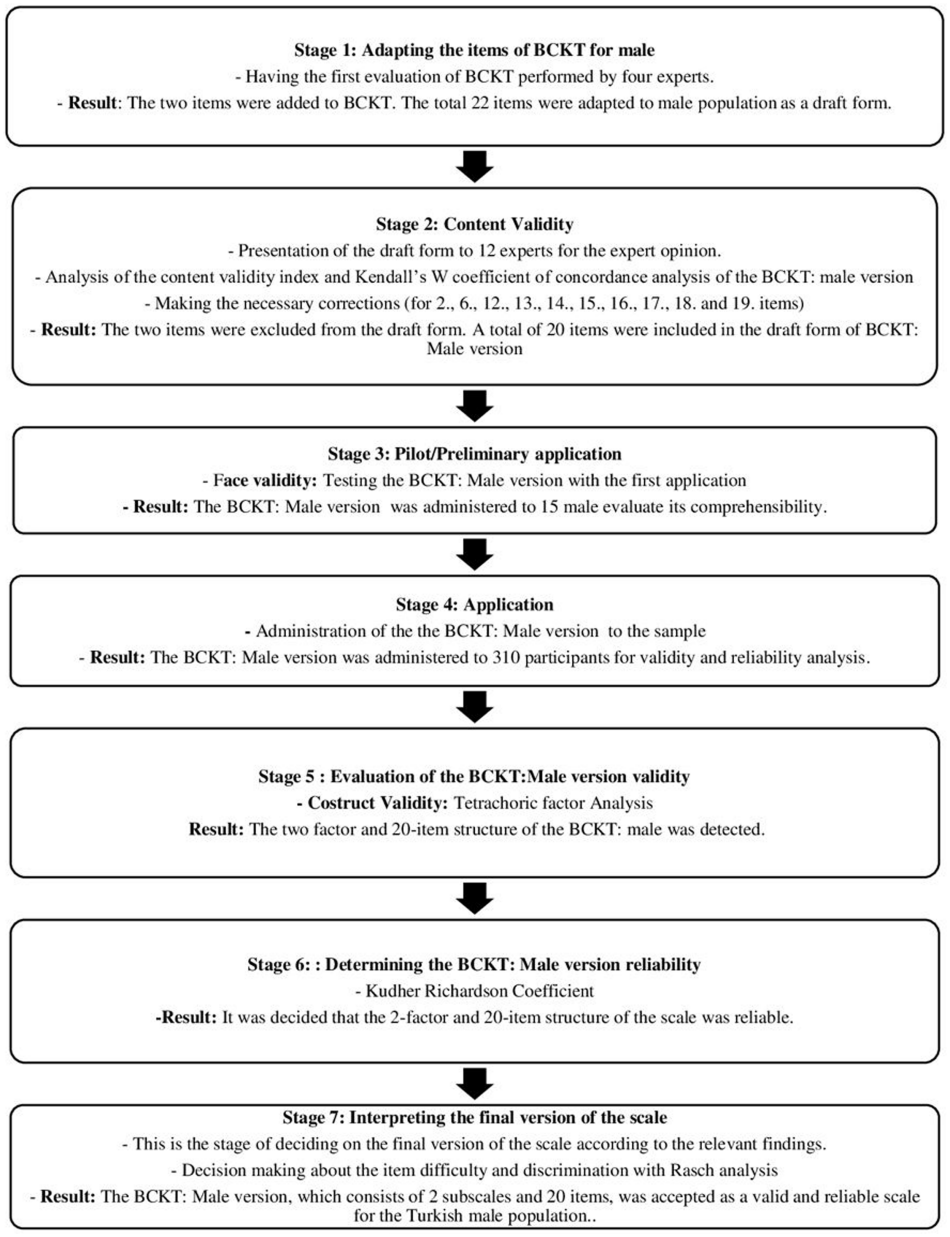
Study flow chart. BCKT, Breast cancer knowledge test.

To enable the application of this instrument to the male population, the adaptation of all items by gender was achieved in seven stages, as illustrated in Figure 1 (39). In the first stage of this process, all items were reviewed by four experts in the field. As a result of this review, it was decided to add two additional items to the scale. Each item was adapted for the male gender, resulting in a draft form consisting of 22 items. The draft form maintained its dichotomous structure, with responses coded as “1” for correct answers and “0” for incorrect or “no ideas” responses.

In the second stage, the draft was submitted to 12 experts for content validity assessment. These 12 experts represent a diverse and highly qualified group with relevant experience and expertise in the field. The experts evaluated the comprehensibility, scope, and cultural appropriateness of the items using a four-point scale: “1 = Not appropriate, 2 = Somewhat appropriate, 3 = Quite appropriate, and 4 = Extremely appropriate.” They also provided additional suggestions. Based on the experts’ feedback, two items (“One in every 100 women in Turkey who experience this condition will develop breast cancer at some point,” “The risk of developing breast cancer in Turkey is higher compared to other regions in Asia and Africa”) from the original BCKT were removed from the draft. The 2nd, 6th, 12th, 13th, 14th, 15th, 16th, 17th, 18th, and 19th items were revised according to their recommendations. Due to the lack of mammography-specific questions and their necessity, two new items (3rd and 11th items) were added in line with expert opinions (“Regular mammograms allow for the early detection of breast cancer in women” and “In our country, as part of the national cancer screening program, women aged 40 to 69 receive free mammograms every two years.”). The “Yes” responses to items 1, 2, 5, 6, 8, 12, 14, 15, 17, 18, 19, and 20 were determined to be incorrect answers. As a result of this expert evaluation, a draft of the 20-item BCKT: Male version was established. The Content Validity Index (CVI) and Kendall’s concordance statistics were subsequently calculated.

In the third stage, a pilot application was conducted to assess the scale’s face validity. During the pilot study, the 20-item draft was administered to 15 male participants. Following the pilot application, it was determined that the scale was comprehensible. The forms obtained from the male participants in this application were excluded from the research.

In the fourth stage, the BCKT: Male version was administered to 310 male participants who met the inclusion criteria for the study. The data was prepared for statistical analysis. In the fifth and sixth stages, validity and reliability analyses of the scale were conducted. In the seventh and final stage, the final form of the scale was interpreted (Figure 1).

### 2.4 Data collection

The data collection form was administered under the researcher’s observation after explaining the purpose of the study to the participants. The administration took place in a quiet and private environment. Participants were asked to mark the options that were most appropriate for them. Questions regarding the completion of the form were answered without any prompting. Filling out the data collection form took approximately 20 min.

### 2.5 Data analysis

The Statistical Package for Social Sciences (SPSS) Version 23.0 was used to analyse the data of the study (IBM Corp., Armonk, NY, USA), Factor 12.04.04 for Windows, and Jamovi version 2.6.13 software. Descriptive statistics for numerical variables were presented as mean ± standard deviation, median, minimum, and maximum, while categorical variables were reported as counts and percentages. The validity of the scale was assessed in terms of content, face, and construct validity. Expert opinions were evaluated on the draft of the BCKT: Male version, using Davis technique to calculate the content validity index and Kendall’s concordance statistic. The normality of the data set was assessed using skewness and kurtosis coefficients (± 2) and the Kolmogorov–Smirnov Test, confirming that the data followed a normal distribution. Tetrachoric Factor Analysis was employed to evaluate construct validity. When working with dichotomous data, tetrachoric correlation is necessary to perform factor analysis in a way that suits the nature of the data. This method allows for the accurate representation of the underlying structures of the data. In the reliability analysis, internal consistency coefficients (Kuder Richardson-20) were calculated. Item difficulty and discrimination indices were tested using Rasch analysis. Rasch analysis is an important tool for determining the validity of a test or scale, especially when working with dichotomous data. This is because it provides a detailed analysis of item fit, item validity, and criterion validity to ensure that each item and participant is accurately evaluated. A significance level of *p* < 0.05 was used.

## 3 Results

### 3.1 Characteristics of the participants

The participants included in the study were registered at the relevant family health center, at least 18 years of age, and had a mother and/or spouse who were alive. Individuals who were healthcare professionals or students in the healthcare field, as well as those whose mother and/or spouse were healthcare professionals, were excluded from the study. All participants were over 18 years old and had a living mother and/or spouse. According to the findings of the study, the average age of the participants was 39.09 ± 11.31. Of the participants, 68.4% were married, 74.2% had a university education or higher, and 93.5% belonged to a nuclear family. Approximately half of the participants (49.7%) perceived their income as equal to their expenses. It was found that 7.1% of the participants had a mother and/or spouse diagnosed with breast cancer, while 2.3% had experienced loss (death) due to breast cancer in their mother and/or spouse. More than half of the participants (57.1%) reported having previously received information about breast cancer and early detection methods in women. Additionally, 26.8% of the participants obtained information about breast cancer and early detection methods from healthcare professionals (Table 1).

**Table 1:** Descriptive statistics of the participants.

Variable	Mean ± SD	Min–Max
Age	39.09 ± 11.31	19–68
Variables	Frequency (N)	Percent (%)
Marital status		
Single	98	31.6
Married	212	68.4
Education level		
Middle School and below	19	6.1
High School	61	19.7
University and above	230	74.2
Family structure		
Nuclear	290	93.5
Extended	20	6.5
Income status		
Income less than expenses	64	20.6
Income equal to expenses	154	49.7
Income more than expenses	92	29.7
Do you have a spouse, mother, or sister aged 40–69?		
Yes	280	90.3
No	30	9.7
Is there a diagnosed cancer in your mother or spouse?		
Yes	41	13.2
No	269	86.8
Is there a diagnosed breast cancer in your mother or spouse?		
Yes	22	7.1
No	288	92.9
Have you experienced a loss due to breast cancer in your mother or spouse?		
Yes	7	2.3
No	303	97.7
Have you received information about breast cancer and early detection methods previously?		
Yes	177	57.1
No	133	42.9
Sources of information on breast cancer and early detection methods*		
Health professional	83	26.8
Internet	78	25.2
Friends	44	14.2
Books and scientific publications	34	11.0
Television	15	4.8

Max, maximum value; Min, minimum value; SD, standard deviation. *n folded.

### 3.2 Validity findings

The validity analysis results, including content and construct validity, indicate that the validity of this instrument is robust.

#### 3.2.1 Content validity

For content validity, expert evaluations were requested. The authors do not know the experts, and a survey form was sent via email to 20 experts interested in the topic. 12 of them responded. Before the evaluation, the purpose of the study, the content of the knowledge test, and its current usage were explained to the experts. The experts were then asked to rate each item on a scale of 1 to 4, based on their knowledge and experience, while also considering social characteristics. Based on the expert opinions obtained, and to mitigate potential bias while ensuring the integrity of item difficulty levels, the CVI analyses were carried out by a statistical consultant rather than the authors. Based on the evaluations from 12 experts regarding the scale, two items were modified and subsequently assessed. After these modifications, the Content Validity Index (CVI) was calculated to be 0.96. Additionally, to evaluate the agreement among the experts, Kendall’s W coefficient of concordance analysis revealed a W value of 0.221 with a *p* value of 0.001.

#### 3.2.2 Construct validity

To evaluate the appropriateness of the sample size for factor analysis, Bartlett’s statistic was found to be 3471.1 (df = 190; *p* = 0.001), and the Kaiser-Meyer-Olkin (KMO) test yielded a value of 0.92, indicating a very good fit. To confirm the suitability of structuring the scale with 20 items and two subscales, a Tetrachoric Factor Analysis was conducted. It was determined that the scale comprised two subscales: risk factors and treatability, as well as risk groups. The Risk Factors subscale, on the other hand, examines knowledge of potential factors that may contribute to the development of breast cancer. These factors include genetic, environmental, and individual characteristics. The Treatability subscale assesses knowledge related to the treatability of breast cancer. It evaluates participants’ understanding of the early detection and treatability of breast cancer. The primary focus of this subscale is the effectiveness of early diagnosis, surgical intervention, and treatment methods. According to the results of the tetrachoric factor analysis, the scale items have been rearranged (Table 2). The fit indices obtained after the tetrachoric factor analysis were within valid ranges and were assessed as having an “excellent fit” (Table 3).

**Table 2:** Distributions of factors’ eigenvalue, variance and factor loadings in tetrachoric factor analysis.

Factors	Items	Eigenvalue	Cumulative variance (%)	KR-20 reliability	Factor determinacy index	Inter-factors correlation
Factor 1	1, 2, 5, 6, 8, 12, 13, 14, 15, 16, 17,18, 19, 20	10.341	51.70	0.95	0.98	0.564
Factor 2	3, 4, 7, 9, 10, 11	1.85	60.94	0.92	0.96	

Items		Factor 1	Factor 2
Item 20	Even with early detection and treatment, it is unlikely for a woman diagnosed with breast cancer to have a normal life expectancy.	0.880	
Item 14	When a cancerous mass in the breast begins to cause pain, it is often too late for successful treatment.	0.844	
Item 19	Even when breast cancer is detected at a very early stage, the chances of successful treatment improve significantly only when the entire breast tissue is surgically removed.	0.821	
Item 6	Women who do not have any known risk factors for breast cancer are never diagnosed with the disease.	0.818	
Item 15	Breast cancer cannot be effectively treated unless the lymph nodes surrounding the breast and located in the armpit are surgically removed.	0.812	
Item 18	If a cancerous mass in a woman’s breast can be felt by hand, it is often too late for effective treatment.	0.806	
Item 17	A woman with a family history of breast cancer has a lower likelihood of being treated successfully compared to a woman without such a family history.	0.802	
Item 2	Wearing a tight-fitting bra that exerts constant pressure on a woman’s breast may contribute to the development of breast cancer over time.	0.686	
Item 1	A severe impact to the breast may increase a woman’s risk of developing breast cancer later in life.	0.667	
Item 5	A woman who gives birth to her first child before the age of 30 has a higher likelihood of developing breast cancer compared to a woman who has her first child after the age of 30.	0.604	
Item 12	Most of the masses found in the breast are cancerous.	0.575	
Item 8	Women over the age of 70 rarely develop breast cancer.	0.465	
Item 16	Breast cancer can sometimes be successfully treated through the surgical removal of the mass and radiation therapy.	0.427	
Item 13	Nowadays, many women are successfully treated for breast cancer without the need for surgical removal of the breast.	0.415	
Item 7	Some fibrocystic breast conditions (non-cancerous masses) increase a woman’s risk of developing breast cancer.		0.865
Item 9	Breast cancer is more commonly observed in women aged 65 compared to those aged 40.		0.787
Item 10	Breast cancer is one of the most common types of cancer among women.		0.702
Item 4	Being overweight can increase the risk of breast cancer in women.		0.677
Item 11	In our country, as part of the national cancer screening program, women aged 40 to 69 receive free mammograms every 2 years.		0.658
Item 3	Regular mammograms allow for the early detection of breast cancer in women		0.564

**Table 3:** Goodness of fit indices values according to tetrachoric factor analysis.

Fit indices	Goodness of fit indices	Calculated values	Compliance level
*χ^2^*/df	0 ≤ χ2/ df ≤ 2	2.592	Excellent fit
NNFI	0.979 ≤ NNFI ≤ 0.996	0.991	Excellent fit
CFI	0.983 ≤ CFI ≤ 0.997	0.993	Excellent fit
RMSEA	0.043 ≤ RMSEA ≤ 0.050	0.047	Excellent fit
RMSR	0.067 ≤ RMSR≤0.095	0.081	Excellent fit

CFI, comparative fit index; NNFI, non-normed fit index; RMSEA, root mean square of approximation; RMSR, root mean square of residuals; *χ*^2^/df, chi-square/degree of freedom.

### 3.3 Reliability findings

The reliability analysis results indicate that the instrument demonstrates good reliability. Following the factor analysis, the Kuder Richardson-20 reliability coefficients were determined to be 0.95 for the first subscale, 0.92 for the second subscale (Table 2), and 0.89 for the overall scale.

#### 3.3.1 Item difficulty and discrimination

**Figure 2:**
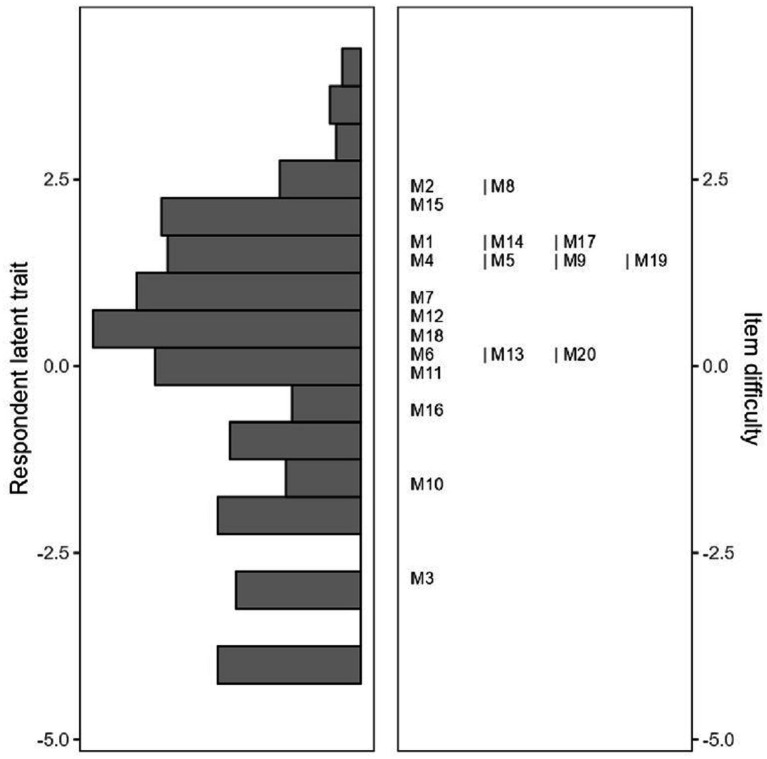
Breast cancer knowledge test: male version item difficulty status and respondent latent trait levels.

According to the Rasch analysis, the model fit statistics were significant, and person reliability was found to be 0.84 (*p* = 0.001) (Table 4). The unweighted mean of squares (outfit) and weighted mean of squares (infit) values for the items ranged from 0.655 to 1.398 (Table 5). Since these values fall within the 0.5–1.5 range, they indicate that the test generally provides a good fit and that both parameters align well with the model. Items 2 and 8 were determined to be the most difficult for participants, followed by item 15. The easiest items were identified as items 3 and 10 (Figure 2, Table 5). Additionally, as shown in Figure 2, items 12 and 18 were assessed as having the highest ability for participants to respond, while item 16 was found to be the item with the lowest response ability among the participants.

**Table 4:** Rasch model fit statistics.

	Min - Max	Mean ± SD	Median	Person reliability	MADaQ3	*p*
Scale	0–19	7.94 ± 5.17	8.0	0.84	0.0772	0.001

MADaQ3, Mean of absolute values of centered Q_3 statistic with *p* value obtained by holm adjustment.

**Table 5:** Item statistics in rash analysis.

	Proportion (%)	Item difficulty	SE measure	Infit	Outfit
M1	24.8	1.7513	0.155	1.163	1.398
M2	15.8	2.5104	0.177	1.044	1.387
M3	85.8	−2.7678	0.200	0.987	1.094
M4	30.3	1.3621	0.148	1.302	1.394
M5	29.4	1.4284	0.149	0.885	0.781
M6	51.0	0.0459	0.143	0.902	0.873
M7	36.8	0.9377	0.144	1.201	1.137
M8	17.7	2.3289	0.171	0.999	0.799
M9	30.0	1.3841	0.148	1.269	1.310
M10	75.2	−1.6820	0.167	0.969	0.932
M11	51.3	0.0255	0.143	1.066	1.026
M12	41.0	0.6722	0.142	0.996	0.884
M13	8.1	0.2285	0.142	0.895	0.842
M14	26.8	1.6098	0.152	0.900	0.696
M15	19.0	2.2149	0.167	0.950	0.703
M16	61.6	−0.6508	0.149	0.802	0.656
M17	27.1	1.5868	0.152	0.978	0.789
M18	43.2	0.5308	0.142	0.816	0.655
M19	28.7	1.4731	0.150	1.028	0.835
M20	50.6	0.0663	0.143	0.880	0.789

Infit, information-weighted mean square statistic; Outfit, outlier-sensitive means square statistic.

As another indicator of the scale’s discriminability, the difference in scores between the lowest 27% of participants and the highest 27% was examined. The analysis revealed a significant difference between the two groups (*p* < 0.01) (Table 6).

**Table 6:** Comparison of the lowest-scoring 27% group with the highest-scoring 27% group (*n* = 168).

Group	Min	Max	Mean ± SD	*t*	*p*
Lowest scoring 27% group (*n* = 84)	0	4	1.50 ± 1.24	11.286	0.001
Highest scoring 27% group (*n* = 84)	12	19	14.48 ± 1.94		

*t*, Independent Sample *t* Test.

## 4 Discussion

In this study, the Breast Cancer Knowledge Test (BCKT), which measures knowledge levels regarding female breast cancer, was adapted for the male population. Previous studies have shown the importance of breast cancer knowledge in promoting early diagnosis and better health outcomes, particularly in women. However, the adaptation of such a scale for men is crucial as awareness around male breast cancer is often limited. For instance, the studies (12, 15, 24, 26) highlighted the lack of breast cancer knowledge in men, which impacts their ability to detect early signs of the disease. In the cultural context of Turkey, where patriarchal values often shape family dynamics, men may play a significant role in influencing women’s participation in breast cancer screening programs. The societal expectations of male authority and decision-making within households can limit women’s autonomy in seeking health care, including early detection and preventive measures for breast cancer. For example, the necessity for both women and their partners to disclose their breasts, a private organ, to a female doctor or healthcare professional during breast cancer screenings can sometimes act as a barrier to participation in screening programs (17, 18). Therefore, adapting the BCKT for male awareness is crucial, as it can help identify how male knowledge and attitudes may affect women’s health-seeking behaviors and participation in these critical health programs. By adapting the BCKT for the male population, this study adds a new dimension to the existing literature by offering a measurement tool that can assess male knowledge and contribute to targeted educational programs.

Content validity assesses whether the scale and its subscales adequately measure the desired variables and evaluates the semantic consistency between concepts. The 20 items developed for the female version of the BCKT were initially reviewed by four experts, and the sentence structures of the items were designed to be appropriate for responses from the male population. The experts decided to add two new items related to breast cancer screening to the scale draft. These items were: “Women who have regular mammograms allow for early diagnosis of breast cancer” and “In our country, as part of the national cancer screening program, women aged 40 to 69 receive mammograms free of charge every two years.” The 22-item draft form of the BCKT was presented to experts to ensure its content validity. To obtain objective results in calculating content validity, the quality and number of experts (ranging from 5 to 40) are of great importance, so opinions were sought from 12 academicians who are experts in the field (41). According to expert opinions, two items related to the prevalence of breast cancer were removed from the draft due to the possibility of changes in prevalence over time. The two items included in the original scale were removed from the scale in this study. These items were: “One in ten women living in Turkey will be diagnosed with breast cancer at some point in their lives” and “The risk of breast cancer for women in Turkey is higher compared to those in Asia and Africa.” The two newly added items to the scale draft were approved by the experts. The inclusion of items such as breast cancer screening was consistent with findings by Okafor et al. (30), who emphasized the need for education on early screening methods to improve outcomes. The expert-approved additions to the scale underscore the relevance of these items in addressing gaps in male awareness. It has been reported that the calculated Content Validity Index (CVI) for a scale should be at least 0.80 to accept content validity (42). In this study, the CVI value calculated for the BCKT adapted for the male population was found to be quite high, indicating sufficient content validity.

To explain the factor structure of the BCKT, a measurement model was evaluated using tetrachoric factor analysis. The tetrachoric factor analysis and high internal consistency measures found in this study are also supported by findings in similar research (43), which demonstrated the effectiveness of using factor analysis to refine measurement tools for cancer knowledge. Tetrachoric factor analysis is used to examine the interaction between two ordinal or dichotomous variables. This analysis aims to uncover an underlying continuous distribution behind two different dichotomous variables (44). In the tetrachoric factor analysis conducted in this study, it was found that all items in the two subscales (risk factors and treatability, risk groups) had factor loadings above 0.40, and the model fit indices were at good or acceptable levels (45).

The Kuder–Richardson-20 (KR-20) reliability coefficient is another internal reliability coefficient evaluated in addition to factor analysis for internal consistency. It assesses the internal consistency of scale items that have dichotomous coding (46). In this study, the internal consistency of the scale items was evaluated as highly reliable since the KR-20 coefficients were above 0.80 for both the total scale and the subscales.

The Rasch analysis is recognized as one of the most powerful methods within the theoretical framework of psychometrics. It examines scales and items in depth and has the potential to establish the highest quality standards for measurement (47). In this study, the items constituting the scale were evaluated dichotomously (0–1), with each item having a correct response. Therefore, the dichotomous Rasch model based on “Item Response Theory” was utilized (48–50). It is recommended that the infit and outfit criteria for items fall between 0.5 and 1.5 for model-data fit (48). These statistics are calculated separately for each item and, when considered together, provide insights into the consistency of responses given to the items (50). In this study, there was an excellent fit between the model and the data, and the Rasch reliability was quite high (51).

The item discrimination index enables the differentiation of individuals based on their scores. To assess whether items are discriminative, the discrimination index must exceed 2 (48). The fact that all items on the scale had discrimination index values above this threshold indicated that it could effectively distinguish between individuals with low and high knowledge levels. The Rasch model can rank all BCKT items from the simplest to the most difficult, measuring which information participants were least likely to answer correctly or which they answered correctly most frequently. The increasing difficulty of the BCKT items is represented by positive and rising logit values, while easier items are represented by negative and decreasing logit values (52). The data of the study indicated that participants struggled the most with items 2 and 8, which misleadingly questioned whether wearing a tight bra causes cancer and the age at which one is likely to develop breast cancer. Similarly, the high difficulty index of item 15, which was related to treatment options for cancer, indicated that respondents had to select “no” to reach the correct answer, underscoring its misleading nature. The literature also reports low correct response rates for similar questions (26, 27). The items that participants found easy to answer were items 3 and 10, which include fundamental, commonly known information, such as that mammography is an early screening method for breast cancer and that breast cancer is a frequently occurring type of cancer (29). In conjunction with this, the difference in scale scores between the high-scoring upper group and the low-scoring lower group in the study was considered to support the discriminative property of the items. In addition to the psychometric evaluation of the adapted Breast Cancer Knowledge Test (BCKT) for the male population, it is crucial to consider the cultural and societal context in which this scale will be implemented, particularly in patriarchal societies. In such settings, traditional gender norms often shape health behaviors and attitudes, with men generally less engaged in health-related topics that are perceived as ‘female concerns,’ such as breast cancer. This can create significant barriers to raising awareness and encouraging screening among men, even though breast cancer, although rarer, can affect them (15, 30).

## 5 Conclusion

According to the results obtained from the adaptation study of the BCKT for adult men, the BCKT: Male version could be utilized as a valid and reliable tool for measuring the knowledge levels of the male population regarding breast cancer, which is commonly observed in women. The adapted BCKT is of great importance in assessing the level of knowledge in ensuring men’s participation in breast cancer awareness and in determining the initiatives taken for the public. The existence of this scale has highlighted the need to enhance knowledge levels about breast cancer, leading to the development and implementation of various strategies. It is well known that one of the barriers to the use of early detection methods for breast cancer is the influence of male peers. Additionally, the lack of an objective measurement tool for assessing men’s knowledge about breast cancer underscores the uniqueness of this study. This adapted tool for the male population would assist in identifying areas where men’s knowledge is lacking and in framing strategies aimed at improving their knowledge levels. In future studies, establishing the knowledge level of men regarding breast cancer in women on an objective basis could address the need for health education and create a roadmap for their mediating role in cancer prevention, early diagnosis, and screening efforts. Therefore, future research should explore the applicability of the adapted BCKT in different cultural contexts to assess whether similar results can be obtained or if modifications are needed to better fit local norms and practices.

## 6 Implications for nursing practice

The scale, adapted for use with a male population, aimed to objectively identify areas where men lack knowledge about female breast cancer, thereby aiding in the development of targeted strategies to enhance their understanding. This adaptation can facilitate increased awareness and participation in breast cancer screening initiatives. With the knowledge test obtained in this study, the effect of men’s knowledge levels about breast cancer on women’s participation in breast cancer screening programs can be measured objectively. In addition to the psychometric evaluation and the identification of knowledge gaps, the implementation of the results from this study is supported by the author’s commitment to sharing these findings with policymakers. This willingness ensures that the study’s outcomes can directly contribute to informed decision-making and the development of relevant health policies, which could facilitate the adoption of more targeted strategies for breast cancer awareness and prevention. In addition, it is recommended that future studies conduct a re-test analysis. This would allow for the assessment of the scale’s temporal stability.

## 7 Strengths and limitations

This study presents several key strengths that contribute to its value in advancing breast cancer awareness among men. First, the adaptation of the Breast Cancer Knowledge Test (BCKT) for the male population is a novel approach, providing an objective means to assess areas where men may lack crucial knowledge about female breast cancer. Another notable strength is the potential for this adaptation to play a significant role in increasing men’s awareness of breast cancer and encouraging their participation in breast cancer screening initiatives. Additionally, the knowledge test developed in this study provides an opportunity to objectively measure how men’s knowledge levels impact the participation of women in breast cancer screening programs. Overall, the study’s design and findings provide a valuable contribution to the literature, offering a reliable tool to assess male breast cancer knowledge and providing insights into potential strategies for enhancing public health initiatives.

While this study provides valuable insights into the adaptation and validation of the Breast Cancer Knowledge Test (BCKT) for the male population, several limitations should be acknowledged. First, the data collection relied on self-reported responses, which can be subject to social desirability bias or inaccurate recall. Participants may have overestimated their knowledge or provided answers they believe are socially acceptable, especially in a topic like breast cancer that can be perceived as a sensitive or gendered issue. Second, this study was conducted within a specific cultural context, and the findings may not be generalizable to male populations in other cultural settings. Third, due to the unwillingness of the participants, the test–retest analysis was not conducted. Due to this condition, the assessment of the scale’s reliability over time is limited.

## Data Availability

The raw data supporting the conclusions of this article will be made available by the authors, without undue reservation.
