# Supplementary material for: A psychometric analysis for the adaptation of the comprehensive breast cancer knowledge test for the male population: a methodological study
Source: Front Public Health. 2025 Jul 23;13:1535564. doi: 10.3389/fpubh.2025.1535564 (PMC12325193; doi:10.3389/fpubh.2025.1535564)
Supplement: Supplementary file 1 [file Table_1.docx]

| **THE BREAST CANCER KNOWLEDGE TEST: MALE VERSION**  **(Adapted Scale and Answer Key)** | | | | |
| --- | --- | --- | --- | --- |
| **No.** | **Items** | **Yes** | **No** | **No idea** |
| **1.** | A severe impact to the breast may increase a woman's risk of developing breast cancer later in life. | 0 | **1**  **(Correct)** | 0 |
| **2.** | Wearing a tight-fitting bra that exerts constant pressure on a woman's breast may contribute to the development of breast cancer over time. | 0 | **1**  **(Correct)** | 0 |
| **3.** | Regular mammograms allow for the early detection of breast cancer in women | **1**  **(Correct)** | 0 | 0 |
| **4.** | Being overweight can increase the risk of breast cancer in women. | **1**  **(Correct)** | 0 | 0 |
| **5.** | A woman who gives birth to her first child before the age of 30 has a higher likelihood of developing breast cancer compared to a woman who has her first child after the age of 30. | 0 | **1**  **(Correct)** | 0 |
| **6.** | Women who do not have any known risk factors for breast cancer are never diagnosed with the disease. | 0 | **1**  **(Correct)** | 0 |
| **7.** | Some fibrocystic breast conditions (non-cancerous masses) increase a woman's risk of developing breast cancer. | **1**  **(Correct)** | 0 | 0 |
| **8.** | Women over the age of 70 rarely develop breast cancer. | 0 | **1**  **(Correct)** | 0 |
| **9.** | Breast cancer is more commonly observed in women aged 65 compared to those aged 40. | **1**  **(Correct)** | 0 | 0 |
| **10.** | Breast cancer is one of the most common types of cancer among women. | **1**  **(Correct)** | 0 | 0 |
| **11.** | In our country, as part of the national cancer screening program, women aged 40 to 69 receive free mammograms every two years. | **1**  **(Correct)** | 0 | 0 |
| **12.** | Most of the masses found in the breast are cancerous. | 0 | **1**  **(Correct)** | 0 |
| **13.** | Nowadays, many women are successfully treated for breast cancer without the need for surgical removal of the breast. | **1**  **(Correct)** | 0 | 0 |
| **14.** | When a cancerous mass in the breast begins to cause pain, it is often too late for successful treatment. | 0 | **1**  **(Correct)** | 0 |
| **15.** | Breast cancer cannot be effectively treated unless the lymph nodes surrounding the breast and located in the armpit are surgically removed. | 0 | **1**  **(Correct)** | 0 |
| **16.** | Breast cancer can sometimes be successfully treated through the surgical removal of the mass and radiation therapy. | **1**  **(Correct)** | 0 | 0 |
| **17.** | A woman with a family history of breast cancer has a lower likelihood of being treated successfully compared to a woman without such a family history. | 0 | **1**  **(Correct)** | 0 |
| **18.** | If a cancerous mass in a woman's breast can be felt by hand, it is often too late for effective treatment. | 0 | **1**  **(Correct)** | 0 |
| **19.** | Even when breast cancer is detected at a very early stage, the chances of successful treatment improve significantly only when the entire breast tissue is surgically removed. | 0 | **1**  **(Correct)** | 0 |
| **20.** | Even with early detection and treatment, it is unlikely for a woman diagnosed with breast cancer to have a normal life expectancy. | 0 | **1**  **(Correct)** | 0 |
